# Associated Factors of Quality of Life in Prison Officers, Brazil

**DOI:** 10.3390/ijerph17103508

**Published:** 2020-05-17

**Authors:** Cristiane S. C. Araújo, Ruth Minamisava, Marcos A. Matos, Camila C. F. Vieira, Priscila V. O. Vitorino, Dolors Rodríguez-Martín, Neuma Chaveiro, Lizete M. A. C. Oliveira, Virginia V. Brasil, Douglas J. Nogueira, Leila A. Salha, Maria A. Barbosa

**Affiliations:** 1 Faculty of Nursing, Federal University of Goias, Rua 227 Qd. 68 s/n, Setor Leste Universitário, Goiania 74605-080, Brazil; minamisava@gmail.com (R.M.); camila_canhete03@hotmail.com (C.C.F.V.); lizete.malagoni@gmail.com (L.M.A.C.O.); viscondebrasil@gmail.com (V.V.B.); dougdeni@yahoo.com.br (D.J.N.); maria.malves@gmail.com (M.A.B.); 2 School of Social Sciences and Heath, Pontifical Catholic University of Goias, Goiania 74690-900, Brazil; fisioprivitorino@gmail.com; 3 Faculty of Medicine Health Sciences, School of Nursing, Universitat de Barcelona, 08028 Barcelona, Spain; dolorsrodriguezmart@ub.edu; 4 Faculty of Languages, Federal University of Goias, Goiania 74690-900, Brazil; neumachaveiro@gmail.com; 5 Faculty of Pharmacy, Federal University of Goias, Goiania 74690-900, Brazil; leilasalha@gmail.com

**Keywords:** prison, quality of life, occupational health

## Abstract

This study analyzed factors associated with the quality of life (QoL) of prison officers (POs) in the Midwest Region of Brazil. POs in five penitentiary units participated in this cross-sectional study. Sociodemographic data were obtained through face to face interview and a World Health Organization Quality of Life abbreviated version (WHOQOL-BREF) questionnaire was applied to assess QoL. Student’s *t*-test or ANOVA were used for bivariate analysis and multiple linear regression was applied for adjusted analysis. The domains used for outcomes were: physical, psychological, social relations, and the environment. The lowest score among WHOQOL-BREF domains was environment (59.9; 95%CI 58.0–61.5). After adjustment, the factors associated with the physical domain were ‘female sex’ and ‘no history of workplace PO-PO violence’; factors associated with the psychological domain were ‘female sex’, ‘without spouse’, and ‘no history of inmate-PO violence’; factors associated with the social relationships domain were ‘female sex’, ‘work experience in years’, ‘no higher education’, ‘no private health insurance’, and ‘no history of inmate-PO violence’; and factors associated with environment domain were ‘female sex’, ‘work experience in years’, ‘no private health insurance’, and ‘no history of PO-PO violence’. This study showed that female workers and those with a history of violence at work had worse QoL scores. This investigation highlights the importance of prison management in promoting QoL of POs, as well as support and development of strategies to prevent workplace violence.

## 1. Introduction

Prison Officers (POs) are responsible for the safety, custody, protection, and activities of prisoners [1,2,3], as well as their discipline and resocialization [4,5]. Studies of these workers show that they suffer job dissatisfaction, stress at work [2,6,7,8,9,10], harassment [11,12], and violence [13,14].

The prison environment can be more adverse than that of many other occupations and is considered high risk [11,15,16,17,18,19]. One of the factors that contribute to hostility and conflicts in the prison environment is the increasing prison population in numbers worldwide. Since 2000, the total world prison population has grown by almost 25%, with the exception of Europe’s, which has decreased by 22%. Oceania increased by 86%, the Region of the Americas by 41%, Asia by 38% and Africa by 29%. In South America, the increase was 175% [20]. In 2017, in Brazil, there were 1507 prison units (almost all of these prison units are governmental) with room for 423,242, but the number of prisoners in these prison units was 726,354. Despite the occupancy rate of 171.62%, the prison population has been growing at a rate of approximately 6% per year [21]. Additionally, in Brazil as well as in other countries there are other aggravating conditions such as an increase in inmate members of organized crime, unsafe working conditions, and riots [22,23,24].

Most studies of POs focus on work related stress. In 2004, a meta-analysis conducted in Canada analyzed the predictive factors of work-related stress in POs and concluded that factors intrinsic to the performance of work, such as the perceived danger of the prison environment by prison employees, were strong predictors of stress in this population [10]. In 2013, a systematic review analyzed eight studies with POs, showing that the organizational structure and climate were predictors of stress and burnout [7]. Another review of the literature on POs was published in 2016 and showed that most studies were about work-related stress, and highlighted the following factors: high workload, overcrowding, and fear [6]. More recently, a systematic review with meta-analysis [25] analyzed 172 articles published from 1980 to 2017 and highlighted the ten most frequent themes in studies of POs: job satisfaction (37 studies), work-related stress (34 studies), organizational commitment (30 studies), turnover (21 studies), emotional exhaustion (18 studies), rehabilitation guidance (16 studies), depersonalization (15 studies), personal fulfillment (12 studies), punitive orientation (11 studies) and mental health (9 studies). Marques et al. [26] in a systematic review noted a gap in investigations into POs that addressed violence and harassment at work

The welfare of correctional officers is a concern due to the conflicts and hostility present in the prison environment [27,28,29], with the potential to negatively impact the QoL of these workers. In fact, POs are in frequent contact with prisoners and are among the professional categories that present risks to physical and psychological well-being along with an unsafe environment [19,29,30,31]. QoL is a multidimensional construct that includes the physical health, psychological health, and social relationships of individuals as well as their environment, and is affected by individual beliefs, experiences, and expectations [32,33]. The WHO developed an instrument to evaluate QoL that is applicable cross-culturally, the WHOQOL-100 [34,35,36,37,38]. A reduced version was later developed, facilitating its large-scale use [39].

In Brazil, one single study on the QoL of POs was conducted in eight prison units in the State of Mato Grosso do Sul, with a descriptive analysis of the QoL life of 110 POs using the World Health Organization Quality of Life abbreviated version (WHOQOL-BREF) instrument. These authors found means of the QoL domains: physical domain (66.89 ± 16.88), psychological domain (68.42 ± 15.25), social relations domain (67.31 ± 18.15) and the environment (58.68 ± 14.26) None of the variables analyzed were related to violence or harassment at work [40].

Assessing the quality life of prison staff can assist in filling the gap in the literature, with the potential of benefit not only for the officers themselves, but also their families and the work environment. Therefore, the objectives of this study were to describe and analyze the factors associated with the QoL of POs in five prison units in the Midwest Region of Brazil

## 2. Materials and Methods 

This was a cross-sectional study of POs working in a large prison complex in the Midwest Region of Brazil, in the City of Goiania, financed by the government of the State of Goiás. The State of Goiás is the most populous state in the region, and the economy is essentially based on agricultural production, livestock, clothing trade, and food and mining industries. This prison complex is composed of five prison units: a closed male prison system; a prison farm; a temporary prison for both genders; a closed female prison system; and a maximum-security unit. This study is part of a larger study which aimed to detect immunological status for hepatitis B infection among POs. This study was approved by the Human and Animal Research Ethics Committee of the Federal University of Goiás (# 2500582/2017). Informed written consent was obtained from each participant. The anonymity and confidentiality of all participants were protected. Data collection occurred during working hours, with prior authorization of the Director General of the prison complex.

All POs working in the five units of the prison complex were eligible for the study. POs who were aware of the results of their immunological tests for Hepatitis B (n = 11) were excluded, along with cases with more than 20% missing data on the WHOQOL-BREF [41].

Sociodemographic data were obtained through interviews and WHOQOL-BREF questions were answered by each PO in a private location in the prison unit from November 2017 to July 2018. Four nurses, previously trained for this purpose, collected all data. The sociodemographic and work related variables were: age in years, gender, race, relationship status, having children, higher education, practicing religion, sexual orientation, private health insurance, work experience in years, workload, history of workplace violence, and history of workplace harassment. A private health insurance variable was included as a proxy variable of socio-economic status, given the great social inequality in Brazil [42].

The WHOQOL-BREF instrument was used to assess QoL and is composed of 26 questions/items. Two items are examined separately: overall QoL and general health. The remaining 24 items are separated into four domains: Physical (composed of seven questions), Psychological (six questions), Social relationships (three questions), and the Environment (composed of eight questions). The answers were rated on a Likert type scale (score from 1 to 5), as described elsewhere (https://www.who.int/mental_health/publications/whoqol/en/) The scores obtained in each domain were scaled into values from 0 to 100. The higher domain score obtained, the better the perception of the individual of their QoL [32,41,43].

Sociodemographic and work characteristics of POs were described. Minimum, maximum mean, and standard deviation, and 95% confidence interval (95% CI) scores of each of the general issues of WHOQOL-BREF (overall quality of life and general health) as well as WHOQOL-BREF domains were calculated. The outcomes of this study were physical, psychological, social, and environmental domains. The scores obtained in each domain were transformed into values from 0 to 100, so that the higher the score obtained, the better the individual’s perception of QoL. The means of the domains were considered statistically different when the 95% CI did not overlap. The Cronbach’s alpha coefficient was calculated for each domain to estimate the reliability of the instrument. Internal consistency is considered excellent when α ≥ 0.9; good 0.8 ≤ α <0.9; acceptable 0.7 ≤ α < 0.8; questionable 0.6 ≤ α < 0.7; poor 0.6 ≤ α < 0.7 and unacceptable α < 0.5 [44].

Student’s *t*-test and ANOVA, when appropriate, were used to detect the differences of the means in the domains of WHOQOL-BREF among strata of the exposure variables. Variables were included for multiple linear regression when *p* < 0.20 in univariate analysis. Multiple linear regression was used to identify the factors associated with the WHOQOL-BREF among POs. All exposure variables were tested for collinearity through tolerance and variance inflation factors. Regression coefficients 95%CI and *p* values are presented in adjusted analysis for each WHOQOL-BREF domain. Values of *p* < 0.05 were considered significant. All analyses were conducted using SPSS software v. 25.0 

## 3. Results

Among 300 eligible POs, 29 (9.7%) refused to participate in the study, and 2 cases had > 20% missing data on WHOQOL-BREF questions. A total of 269 cases were therefore included in this study. The majority of the participants were male, aged 30 years or older, with higher education, with spouse, had private health insurance, had less than five years of working experience, and worked 24 h with a 72-h rest period (Table 1). Workplace inmate-PO violence was reported by 19.3% of PO and 16.5% reported workplace PO-PO violence.

The mean scores of each of the general issues of WHOQOL-BREF (overall quality of life and general health) are presented in Table 2, where it is shown that overall QoL was better perceived by POs than general health.

The domain scores were showed in Table 3. The lowest score among WHOQOL-BREF domains was the environment domain (59.9 ± 14.9).

Table 4 presents the univariate analysis to identify factors potentially associated with the WHOQOL-BREF domains among POs. 

Included in the adjusted analysis were: for the Physical domain, the variables gender and history of workplace PO-PO violence; for the Psychological domain, the variables gender, age, spouse, children, sexual orientation, private health insurance, history of workplace inmate-PO violence, and history of workplace PO-PO violence; for Social relationships, the variables gender, spouse, higher education, private health insurance, work experience, history of workplace inmate-PO violence, and history of workplace PO-PO violence; finally, for the Environment domain, the variables gender, race, spouse, children, private health insurance, work experience, and history of workplace PO-PO violence.

Table 5 presents the factors associated with the domains of the WHOQOL-BREF, after adjustments. Female sex and history of workplace PO-PO violence were negatively associated with Physical domain. Female participants, without spouse, and history of workplace inmate-PO violence showed negative association with Psychological domain. Female sex, less work experience in years, not higher education, and history of workplace inmate-PO violence were negatively associated with Social relationships domain. Female sex, less work experience in years, without private health insurance, and history of workplace PO-PO violence were negatively associated with Environment domain.

## 4. Discussion

The main objective of this study was to investigate the factors associated with the QoL of POs in a prison complex in Brazil. Among the WHOQOL-BREF domains, the environment domain had the worst score, with a significantly lower mean than the other domains. The factors that contributed to the lowest score in the environment domain were being female, less work experience, not having private health insurance, and history of workplace PO-PO violence. 

Female POs presented worse scores when compared to male POs in all four WHOQOL-BREF domains: physical, psychological, social relations and environment. From our results, it is possible to imagine that the challenges faced by female POs go beyond those faced by other working women. The work of POs is culturally seen as men’s work, since women are physically weaker than men. In fact, in the division of tasks at the organizational level, activities that are “dangerous and that would require greater physical strength” are usually directed to male POs [45]. However, most women worked with female prisoners, and those who worked in male prison units were never responsible for discipline and control of inmates. It is not evident that female POs are less capable, more likely that they perform their jobs using different skill sets. More “delicate”, more affectionate, or more “feminine” women may feel more pressured to have “masculine” attitudes, particularly in hostile environments.

The study that applied the WHOQOL-BREF in the general population in Brazil found that women report worse QoL in most domains [46] reinforcing the idea that they seem to be more emotional and sensitive [47]. Although Cruz et al. [46] studied the general population in Brazil, his study used a population in the South Region of the country and may not faithfully represent the population in the Midwest Region, given the continental dimension and cultural diversity in the country. Therefore, it is difficult to know if these results are applicable only for female POs, and what would qualify as a study due to the absence of a control group composed of the general population.

It is quite possible that the worse perceptions of POs regarding QoL in the environmental domain were certainly influenced by the extreme environment of Brazilian prisons. The prison complex in this study has not avoided prison overcrowding endemic in Brazil [21], a chronic and historical problem in the country and responsible for worsening human rights violations [48].

As a result of prison overcrowding there may be violence among prisoners, and in certain critical situations even unnecessary violence among POs. A study conducted in Australia showed that the perceptions of POs regarding work-related environmental adversities were significantly more pronounced than the perceptions of other workers (except police and emergency service workers). These same POs perceived their work environment as highly threatening, unpredictable, prone to suffering traumatic events and requiring a high level of vigilance and caution in their actions [19]. In Canada, another study with POs reported psychological harassment by both inmates and colleagues, supervisors, and subordinates [11]. In some Latin American countries [49], there are also reports of violence as part of training or as initiation or admission rites for POs.

Particularly in Brazil, violence was observed both between prisoners and officers, and prisoners with each other [23,50], which probably raises great concern among POs. The sustained level of violence inside prisons can make people apprehensive, particularly if these prisons fail to implement serious measures to prevent and manage violence.

Several factors were associated with a lower QoL in the field of social relations: being a woman, less work experience, not having any higher education, and a history of violence among POs. Conflicts at work [51] are brought home to families, with reports of officers having become violent with family members, and others feeling threatened or persecuted. In the work environment and outside of it, fear can be constant, due to the absence of or reduction of physical security and protection, taking into account not only their own safety, but also that of family members [45].

Prison violence happens for a variety of reasons, including drugs, bullying, debts, vulnerabilities, or bad relationships with POs. Prison violence can also be caused by individuals with a tendency toward violence, precarious environments, local culture that accepts violence as a solution to difficulties or as a way of establishing respect, boredom, and prisoners who feel mistreated by POs [52].

This study represents exploratory work in a new area, and therefore, several limitations must be taken into account when interpreting the results. Among the limitations of this study, cross-sectional design does not allow the establishing of causal relationships and longitudinal studies would be desirable to analyze the dynamics of violence and QoL over the years. In other words, it is difficult to establish in a cross-sectional study whether violence results in worse QoL or if it is a worse QoL that results in violence. It is also plausible that another condition/variable, such as a culture of “machismo” or male bravado, for example, could reduce QoL or increase violence at work. Another limitation is that it covered prison units in a single region of the country and these results are not representative of the country as a whole. Future studies involving large samples including other regions and cultures need to be carried out to support these conclusions. The strength of this study lies in the low refusal rate and in exploring the vulnerability and influence of working conditions on the QoL of this group responsible for guarding prisoners. 

Many experiences of reducing violence within prisons are reported in the literature. Brazil, however, still has a culture of protecting society by separating criminals and confining them. Social re-insertion programs still have few successful experiences. In this respect, the results of this study revealed an adverse environment, which affected social relationships of POs. This set of factors that negatively affect the QoL for POs is also relevant for managers of prison institutions and can support future interventions to improve the QoL of this population group. Furthermore, these results provide a baseline for future research.

## 5. Conclusions

The environment was the most affected dimension of QoL for POs and we also found that female workers and those with a history of violence at work had worse scores for QoL. These results highlight the importance of prison unit managers in promoting the QoL of correctional officers, supporting and developing interventions to prevent PO-PO and prisoner-PO violence. This investigation also may contribute to the training of future POs. The development of a more humane prison policy can be beneficial not only for POs, but also for their families, inmates, and society in general.

## Figures and Tables

**Table 1:** Sociodemographic and work characteristics of prison officers (n = 269).

Variables	N	%
Gender		
Male	202	75.1
Female	67	24.9
Age in years		
<30	102	37.9
≥30	167	62.1
Race		
White	75	28.1
Non-white	192	71.9
Spouse		
Yes	147	54.6
No	122	45.4
Children		
Yes	121	45.0
No	148	55.0
Higher Education		
Yes	189	70.5
No	79	29.5
Religion		
Yes	228	85.1
No	40	14.9
Sexual orientation		
Heterosexual	262	97.4
Homosexual	7	2.6
Private health insurance		
Yes	160	59.5
No	109	40.5
Working experience (years)		
<5 years	224	83.3
≥5 years	45	16.7
Workload		
24hours/4 days	204	75.8
40 h/week	55	20.4
30 h/week	10	3.7
History of workplace inmate-PO violence		
Yes	52	19.3
No	217	80.7
History of workplace PO-PO violence		
Yes	44	16.5
No	223	83.5

**Table 2:** Scores of each of the general issues of World Health Organization Quality of Life abbreviated version (WHOQOL-BREF) of prison officers.

General Issues	Minimum	Maximum	Mean	SD	95% Confidence Interval
Overall quality of life	0.0	100.0	73.3	17.7	71.1–75.4
General health	0.0	100.0	68.8	20.4	66.2–71.1

SD—Standard deviation.

**Table 3:** Scores of the World Health Organization Quality of Life abbreviated version (WHOQOL-BREF) domains in prison officers.

Domains	Minimum	Maximum	Mean	SD	95% Confidence Interval	Cronbach Coefficient
Physical	32.1	100.0	74.5	14.1	72.8–76.2	0.784
Psychological	29.1	100.0	74.9	13.1	73.2–76.4	0.726
Social relations	0.0	100.0	75.0	17.1	73.0–77.0	0.689
Environment	18.8	93.8	59.9	14.9	58.0–61.5	0.761

SD—Standard deviation.

**Table 4:** Factors potentially associated with the World Health Organization Quality of Life abbreviated version (WHOQOL-BREF) domains among prison officers.

Variables	Physical Domain			Psychological Domain			Social Relationships Domain			Environment Domain		
	Mean	SD	*p*-Value	Mean	SD	*p*-Value	Mean	SD	*p*-Value	Mean	SD	*p*-Value
Gender												
male	76.3	13.8	0.001	76.1	12.7	0.004	76.7	16.7	0.011	60.9	14.3	0.023
female	69.4	14.0	-	70.8	14.2	-	70.5	18.0	-	56.1	16.1	-
Age												
<30 years	75.1	13.0	0.650	72.7	13.5	0.041	75.2	16.2	0.986	58.6	14.3	0.315
≥30 years	74.3	14.9	-	76.1	13.0	-	75.1	17.8	-	60.4	15.2	-
Race												
white	75.0	12.8	0.822	75.6	10.7	0.450	76.2	12.9	0.455	62.0	12.0	0.082
non-white	74.5	14.7	-	74.4	14.2	-	74.7	18.7	-	58.8	15.9	-
Spouse												
yes	74.5	14.4	0.938	76.7	11.6	0.012	76.6	16.5	0.137	61.1	14.8	0.101
no	74.7	13.9	-	72.5	14.8	-	73.4	17.8	-	58.1	14.9	-
Children												
yes	74.0	15.2	0.554	76.1	13.3	0.146	75.5	16.5	0.768	61.1	15.5	0.183
no	75.1	13.3	-	73.7	13.2	-	74.9	17.8	-	58.6	14.3	-
Higher Education												
yes	74.2	13.7	0.440	74.3	13.3	0.289	73.4	17.0	0.006	60.1	14.4	0.656
no	75.7	15.4	-	76.2	12.9	-	79.6	16.9	-	59.1	16.1	-
Religion												
no	75.3	15.5	0.749	73.0	16.7	0.452	73.5	18.6	0.509	59.9	15.3	0.911
yes	74.5	14.0	-	75.1	12.6	-	75.5	17.0	-	59.6	14.9	-
Sexual orientation												
heterosexual	74.7	14.2	0.550	75.0	13.3	0.161	75.3	17.3	0.446	59.8	14.9	0.701
homosexual	71.4	14.0	-	67.9	9.5	-	70.2	15.1	-	57.6	15.0	-
Private health insurance												
yes	74.2	13.7	0.551	75.7	12.6	0.197	76.9	15.3	0.045	62.6	13.9	0.000
no	75.2	14.9	-	73.5	14.1	-	72.6	19.4	-	55.4	15.3	-
Work experience in years												
<5 years	75.0	13.9	0.237	74.7	13.1	0.888	74.5	16.9	0.160	58.7	14.6	0.010
≥5 years	72.3	15.5	-	75.1	14.2	-	78.4	18.4	-	64.9	15.4	-
Workload												
24 h/4 days	75.0	14.0	0.642	75.0	13.1	0.656	74.4	17.6	0.421	59.6	14.5	0.408
40 h/week	73.0	14.9	-	73.7	13.3	-	77.7	16.4	-	60.0	16.3	-
30 h/week	74.9	12.6	-	77.5	16.4	-	76.7	12.3	-	61.6	17.1	-
History of workplace inmate-PO violence												
yes	72.4	14.8	0.219	71.6	14.5	0.053	70.8	15.9	0.044	57.5	13.4	0.235
no	75.1	14.0	-	75.6	12.9	-	76.2	17.4	-	60.3	15.2	-
History of workplace PO-PO violence												
yes	69.30	16.5	0.021	71.7	13.0	0.097	69.2	16.2	0.014	54.8	14.3	0.016
no	75.58	13.5	-	75.3	13.3	-	76.2	17.2	-	60.7	14.9	

**Table 5:** Factors associated with the World Health Organization Quality of Life abbreviated version (WHOQOL-BREF) domains of prison officers.

Variables	Physical Domain ^a^ R^2^ = 0.068			Psychological Domain ^b^ R^2^ = 0.068			Social relationships Domain ^c^ R^2^ = 0.097			Environment Domain ^d^ R^2^ = 0.100		
	B	95% CI	*p*-Value	B	95% CI	*p*-Value	B	95% CI	*p*-Value	B	95% CI	*p*-Value
Female	−6.57	–10.41; –2.73	0.001	–5.25	–8.89; –1.62	0.005	–6.13	–10.76; –1.51	0.010	–4.10	–8.08; –0.12	0.044
Without a spouse(a)				–3.81	–6.96; –0.67	0.018						
Working experience in years							0.04	0.00; 0.07	0.050	0.03	0.00; 0.07	0.038
College							6.26	1.82; 10.69	0.006			
Without private health insurance							–3.58	–7.80; 0.64	0.096	–6.02	–9.63; –2.41	0.001
Without history of workplace violence prisoner-prison officer				3.55	0.41; 6.69	0.027	5.50	1.39; 9.62	0.009			
Without history of workplace violence prison officer-prison officer	5.91	1.45; 10.38	0.010							4.46	0.61; 8.32	0.023

^a^ Adjusted for: age in years. ^b^ adjusted for: age in years, children, sexual orientation, private health insurance, history of workplace violence prison officer-prison officer. ^c^ adjusted for: age in years, spouse, history of workplace violence prison officer-prison officer. ^d^ adjusted for: age in years, color, spouse, children.
